# Heat Source Parameter Identification Based on Attention-Enhanced Residual Convolutional Neural Network

**DOI:** 10.3390/ma18174174

**Published:** 2025-09-05

**Authors:** Hao Jiang, Xinyu Liu, Zhenfei Guo, Tianlei Yang, Mengyi Chen, Zongzhe Man, Xiao Wei, Jiangfan Zhou, Da Liu

**Affiliations:** 1 College of Mechanical and Electrical Engineering, Northeast Forestry University, Harbin 150040, China; 2 National Frontiers Science Center for Industrial Intelligence and Systems Optimization, Northeastern University, Shenyang 110167, China; 3 Shanghai Institute of Aerospace Systems Engineering, Shanghai 201100, China; 4 Beijing Institute of Strength and Environment Engineering, Beijing 100076, China; 5 School of Mechanical Engineering, Hubei University of Technology, Wuhan 430068, China

**Keywords:** heat source parameters, welding simulation, residual convolutional neural network, parameter inversion

## Abstract

Heat source parameters are critical input variables in welding thermal analysis, directly and significantly affecting the accuracy of the temperature field distribution, welding distortion, and residual stress prediction. This is particularly important in safety-critical welded structures, where high-precision heat source parameter identification is essential for ensuring the thermal simulation accuracy and mechanical performance reliability. Traditional parameter identification methods based on finite element simulations or experiments have limitations in adapting to complex working conditions and variable environments. To address this, this paper proposes the Heat Source Parameter Identification Network (HSPINet) model based on a residual convolutional neural network (ResNet) architecture with an attention mechanism capable of extracting key features from the weld morphology of T-joint structures, while accounting for the influence of process parameters and joint dimensions to achieve efficient and accurate identification of heat source parameters. This study not only enhances the intelligence level of heat source parameter identification but also provides a practical, intelligent tool for welding simulation and thermal field evaluation in complex industrial applications, demonstrating significant theoretical value and broad applicability in laser processing and manufacturing scenarios.

## 1. Introduction

Welding is extensively employed in diverse engineering fields, including construction, aerospace, marine structures, and the automotive industry [1,2]. Computational Welding Mechanics (CWM) has become an essential branch in welding research and engineering applications. With the advancement of computer and numerical simulation technologies, CWM methods have become increasingly diversified [3,4,5]. Among these methods, the finite element method (FEM), based on thermo-elasto-plastic mechanics, remains the primary computational approach, being widely employed to predict the temperature field, residual stress, and distortion in welded joints [6,7]. This method typically adopts a sequentially coupled approach, wherein the temperature field is simulated through thermal analysis based on heat conduction theory and heat source models, which in turn directly affects the resulting residual stresses and distortions. In particular, in critical connection zones where T-joint welds are commonly used, heat input control plays a decisive role in residual stress and fatigue life, making the accurate identification of heat source parameters especially crucial. Therefore, ensuring both the accuracy and precision of heat source parameters is vital to the reliability of thermal field simulations [8].

Methods for determining heat source parameters can be generally categorized into three types: trial-and-error, finite element model updating, and surrogate modeling. The trial-and-error method relies on empirical knowledge and extensive experiments to identify suitable heat source parameters. Raftar et al. employed the finite element software Simufact Welding and applied thermo-elasto-plastic analysis to study the welding deformation and residual stress in cruciform fillet welded joints [9]. By comparing the simulated results of temperature history, weld morphology, deformation, and residual stress with experimental data, the model was validated, and the effects of different plate thicknesses and welding sequences on deformation were analyzed. Yan et al. developed a multi-pass laser welding numerical model for 5A06 aluminum alloy plates with a thickness of 30 mm and validated it using experimental data of molten pool geometry and residual stress [7]. Based on the proposed numerical model, they investigated the effects of interpass cooling time and welding speed on welding behavior. Yan et al. also conducted welding experiments on corrugated steel web I-beams to obtain transverse and longitudinal residual stress data at multiple measurement points [10]. A 3D thermo-elasto-plastic finite element model of the I-beam was developed using Abaqus and validated by comparing the peak temperature and residual stress with experimental results. The distribution of residual stress and deformation was then analyzed. Ghafouri et al. used a 3D thermo-elasto-plastic finite element method based on the double-ellipsoidal heat source model to study the effects of different welding sequences and external constraints on angular distortion and residual stress development in short fillet welds of high-strength steel (HSS) S700 [11]. The accuracy of the numerical model was validated against experimental results for weld morphology, temperature history, welding deformation, and residual stress. However, while such methods can partially satisfy the basic requirements of temperature field simulation, they fall short in providing the comprehensive and accurate optimization of heat source parameters and are constrained by the limitations of researchers’ experience and the high cost of experimental trials.

The finite element model updating method employs numerical simulation techniques such as finite element analysis to replicate weld morphology or temperature history, and it compares the simulation with experimental results to establish a suitable objective function. It then utilizes gradient-based or intelligent optimization algorithms to obtain the optimal heat source parameters, thereby providing a more scientific approach to parameter determination. Walker et al. proposed a novel methodology to determine the optimal element size and thermal finite element model parameters for welding applications, and validated it using experimental test cases involving laser beam welding (LBW) [12]. The calibration procedure was designed to accommodate combinations of various heat source models, allowing its application in common welding processes. Farias et al. proposed an optimization method that used inverse problem techniques combined with genetic algorithms to identify the optimal heat source parameters [13]. This method was validated for butt and lap joints of AISI 1020 carbon steel and AISI 304 stainless steel in Gas Tungsten Arc Welding (GTAW) processes. Jiao et al. defined the deviation in fusion zone shape as the objective function and proposed an interactive computational procedure by integrating numerical simulation with a particle swarm optimization algorithm, achieving efficient automatic parameter calibration [14]. A novel objective function, termed the total fusion zone error, was introduced to quantify the deviation in fusion zone geometry. Although such methods significantly reduce the cost and time of manual trials while improving the accuracy of heat source parameter correction, they require multiple finite element analyses to iteratively update parameters until the numerical results match the experimental data. This process results in high computational costs.

To improve the efficiency of heat source parameter inversion, several surrogate model-based approaches have been proposed. Bai et al. treated the heat source parameters as input variables and the deviation between simulated and experimental welding deformation/residual stress as the response, thereby establishing an equivalent heat source parameter inversion method based on the response surface methodology [15,16]. Jiao et al. derived an analytical solution to the heat conduction equation based on a conical heat source, and subsequently developed a multivariate linear regression model using the molten pool profile and heat input as independent variables and the geometric parameters of the conical heat source as dependent variables [17]. Machine learning methods are capable of capturing nonlinear and complex relationships among high-dimensional physical variables. They offer advantages such as fast computation and ease of implementation, and have also been widely applied in the field of welding [18,19]. However, studies focusing on heat source parameter identification remain relatively scarce. Fu et al. developed a neural network program based on the Levenberg–Marquardt algorithm to invert heat source parameters for the Goldak double-ellipsoidal heat source model, and they applied it to study the welding sequence of fillet welded structures [20,21]. Kitano et al. constructed a convolutional neural network-based model for evaluating heat source parameters, using the relative welding position, weld geometry, and temperature history as inputs [22]. In our previous study, we proposed a deep learning method based on convolutional neural networks for identifying heat source parameters from weld morphology and validated its feasibility using laser butt-welded flat plate cases [23]. Compared to traditional trial-and-error and finite element updating methods, machine learning offers a more efficient means of identifying heat source parameters. However, the deep learning models developed in previous studies were applicable only to specific process parameters and joint dimensions, which severely limits their applicability.

Therefore, there is an urgent need to develop a heat source parameter identification model that is applicable to varying process parameters and joint sizes. Such a model is of great significance for improving the accuracy and efficiency of Computational Welding Mechanics simulations. Firstly, T-joint weld morphology images under various process and heat source parameters were generated using finite element simulations. After data preprocessing, these images were used to construct a dataset for training the HSPINet model. Subsequently, an HSPINet model based on an attention-enhanced residual convolutional neural network (ResNet) architecture was proposed. This model integrates weld images and process parameters to enable the high-precision prediction of heat source parameters. Its performance was validated. This research not only demonstrates the feasibility and robustness of employing advanced deep learning architectures for inverse heat source parameter identification but also provides valuable insights for extending such approaches to broader classes of welding joints and process conditions. In doing so, it offers significant potential for improving the reliability of thermal simulations and the mechanical integrity of welded structures in complex industrial applications.

## 2. Theoretical Analysis

The temperature field *Φ*(*x*, *y*, *z*, *t*) of the welded structure can be obtained through transient thermal analysis by solving the heat conduction equation. It can be expressed as follows [24]:

(1) $$\rho C\frac{\partial \Phi }{\partial t}-\frac{\partial }{\partial x}\left(k_{x}\frac{\partial \Phi }{\partial x}\right)-\frac{\partial }{\partial y}\left(k_{y}\frac{\partial \Phi }{\partial y}\right)-\frac{\partial }{\partial z}\left(k_{z}\frac{\partial \Phi }{\partial z}\right)-Q=0$$

where *ρ* is the density, *C* is the specific heat capacity, and *t* is time. *k_x_*, *k_y_*, and *k_z_* denote the thermal conductivities in the *x*, *y*, and *z* directions, respectively; *Q* = *Q*(*x*, *y*, *z*, *t*) represents the internal heat generation rate of the material. It is typically characterized by using a heat source model to describe its distribution and magnitude.

During the welding process, heat is predominantly dissipated through convection and radiation. The heat loss due to convective heat transfer, *q*_h_, can be calculated by applying Newton’s law of cooling:

(2) $$q_{h}=h_{c}(T-T_{0})$$

where *h*_c_ is the convective heat transfer coefficient, and *T*_0_ and *T* represent the initial and current temperatures of the welded structure, respectively.

The heat loss due to radiative heat transfer, *q*_r_, can be calculated using the Stefan–Boltzmann law:

(3) $$q_{r}=\varepsilon \kappa \left[{\left(T+273.15\right)}^{4}-{\left(T_{0}+273.15\right)}^{4}\right]$$

where *ε* is the emissivity, and *κ* is the Stefan–Boltzmann constant.

As outlined above, predicting the thermal field requires prior knowledge of the geometric shape of the welded structure **G**, the thermophysical properties of the material **ξ**_t_, the thermal boundary conditions **Ω**_t_, the manufacturing process parameters **P**_m_, and the heat source parameters **P**_h_. Under these conditions, the temperature field *Φ* is solved such that it satisfies the heat balance equation, expressed as

(4) $$\phi =F_{P\to \Phi }(\xi_{t},P_{m},P_{h},G,\Omega_{t})$$

Since the weld region is defined as the area where the temperature exceeds the melting point, the weld morphology *Μ* is related to the temperature field *Φ* as follows:

(5) $$M=F_{\phi \to M}(\phi )$$

Substituting Equation (4) into Equation (5) and rearranging gives

(6) $$M=F_{\phi \to M}\left(F_{P\to \phi }(\xi_{t},P_{m},P_{h},G,\Omega_{t})\right)=F_{P\to M}(\xi_{t},P_{m},P_{h},G,\Omega_{t})$$

where *F_Φ_*_→*M*_ represents the mapping from heat source parameters to the temperature field, and *F_P_*_→*M*_ represents the mapping from heat source parameters to weld morphology.

Based on the above equation, the inverse problem of identifying heat source parameters can be described as follows:

(7) $$P_{h}=G_{\mathrm{hsp}}(\xi_{t},P_{m},M,G,\Omega_{t})$$

The concept of identifying heat source parameters involves establishing the mapping relationship *G*_hsp_. Accordingly, the HSPINet model proposed in this section is formulated as follows:

(8) $$P_{h}=G_{\mathrm{hsp}}(\xi_{t},P_{m},M,G,\Omega_{t},\Theta )$$

where **Θ** denotes the structural and weight parameters of the HSPINet model.

In our previous study, we investigated laser butt joints of 6061 aluminum alloy with specific process parameters. In this case, the material’s thermophysical properties **ξ**_t_, process parameters **P**_m_, geometric configuration **G**, and thermal boundary conditions **Ω**_t_ were predetermined. Therefore, Equation (8) can be simplified as

(9) $$P_{h}=G_{\mathrm{hsp}}(M,\Theta )$$

However, the deep learning model derived from Equation (9) has limited generalization to varying process parameters and geometric dimensions and is only applicable to butt joints. Therefore, in this study, we developed an HSPINet model capable of identifying heat source parameters for T-joints with varying process parameters and geometric dimensions. Accordingly, Equation (8) can be simplified as

(10) $$P_{h}=G_{\mathrm{hsp}}(P_{m},M,G,\Theta )$$

## 3. Dataset Construction

### 3.1. Weld Morphology Simulation

#### 3.1.1. Finite Element Model

**Figure 1:**
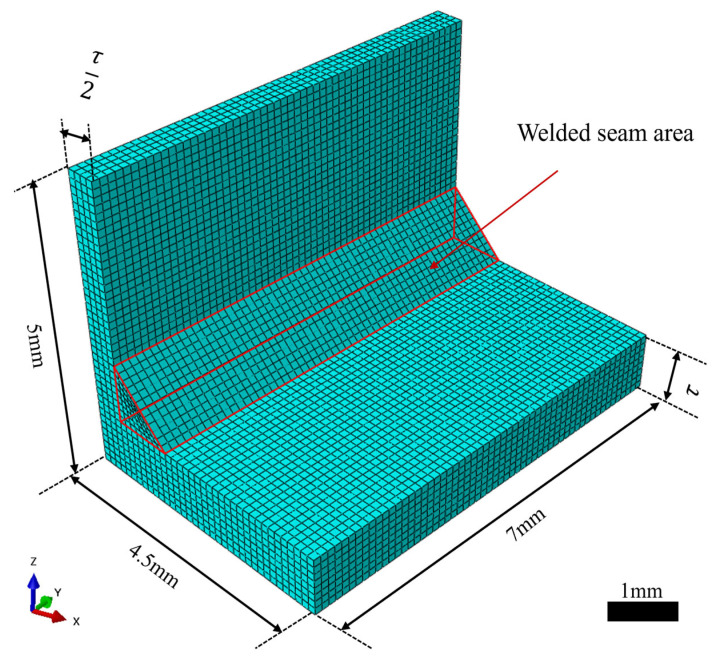
Half-domain finite element model of the double-sided T-joint.

To simulate weld morphology under various process parameters, heat source parameters, and geometric dimensions, a 3D thermal finite element model of a double-sided T-joint was developed in Abaqus (2022). Due to symmetry, only half of the geometry was modeled, and its dimensions are shown in Figure 1. The weld cross-section was defined as an isosceles triangle with a base width of 1 mm and height of 1 mm, extending 7 mm along the length direction. The welding plate thickness (*τ*) was incorporated as one of the input variables within the sample space (1–3 mm) to enhance the model’s generalization capability across different structural dimensions and to assist in the accurate identification of heat source parameters. The mesh was generated using 8-node linear heat conduction solid elements (DC3D8) with an element size of 0.1 mm, as shown in Figure 1.

**Figure 2:**
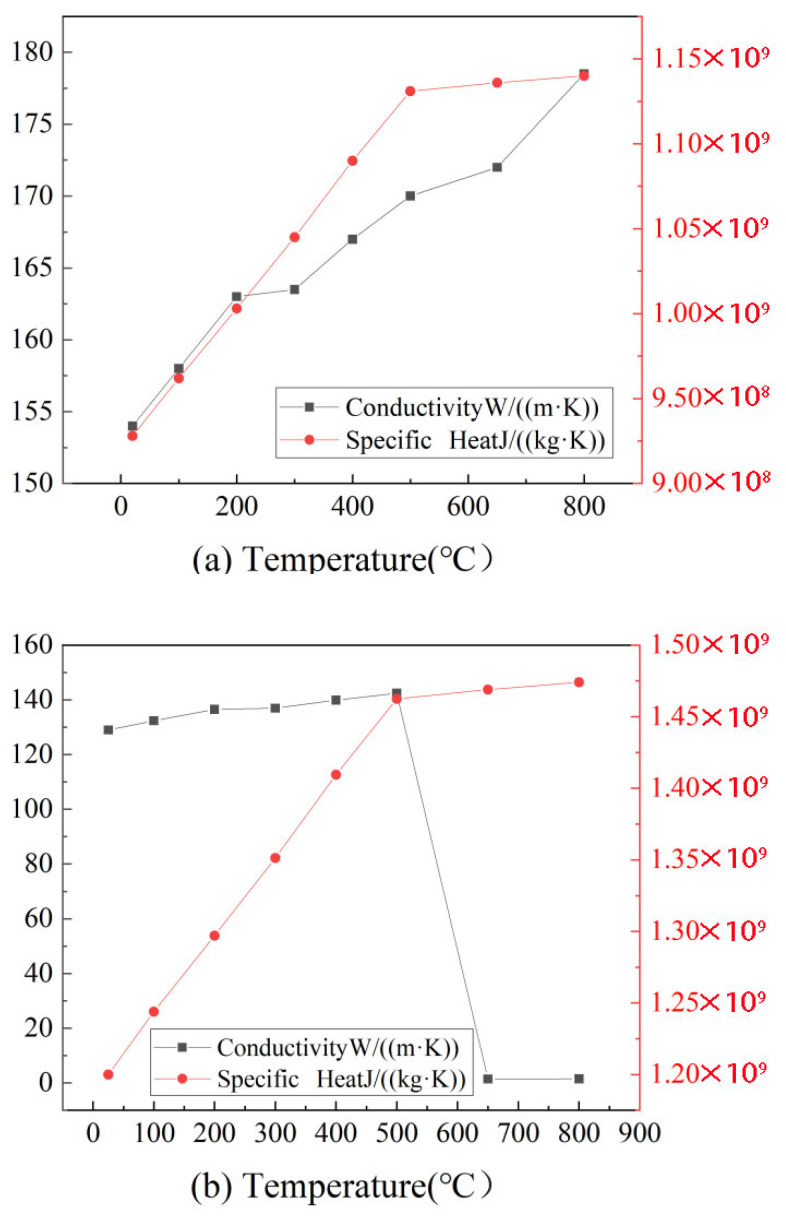
Temperature-dependent thermophysical properties of aluminum alloys in FEM simulations: (**a**) 2060 aluminum alloy, (**b**) 2099 aluminum alloy.

Adiabatic boundary conditions were applied to the symmetry plane, while convection and radiation heat transfer were applied to the other surfaces. The convective heat transfer coefficient was set to vary significantly with the temperature, from 1.366 W/(m^2^·K) at 20 °C to 800.0 W/(m^2^·K) at 3000 °C, reflecting the enhanced forced convection at high surface temperatures resulting from the reduced melt viscosity, thermal buoyancy, and metal vapor recoil. For intermediate temperatures, linear interpolation was applied [25], and the detailed coefficient values are listed in Table 1. The surface emissivity was set to 0.8 [26], as aluminum alloys in the molten state during laser welding typically exhibit emissivity values close to this level. The heat source was applied in the form of surface and volumetric heat flux, with the initial temperature uniformly set to 20 °C. During welding, thermal cycling leads to continuous changes in the material’s chemical composition, microstructure, and dislocation density, which in turn causes the material properties to vary with temperature during welding. Therefore, to improve simulation accuracy, temperature-dependent thermophysical properties of the material were incorporated into the model [27]. The thermophysical properties are shown in Figure 2. Since the variation above the melting point is relatively small, material properties were defined up to 800 °C, and linear extrapolation was applied beyond this temperature [28].

**Figure 3:**
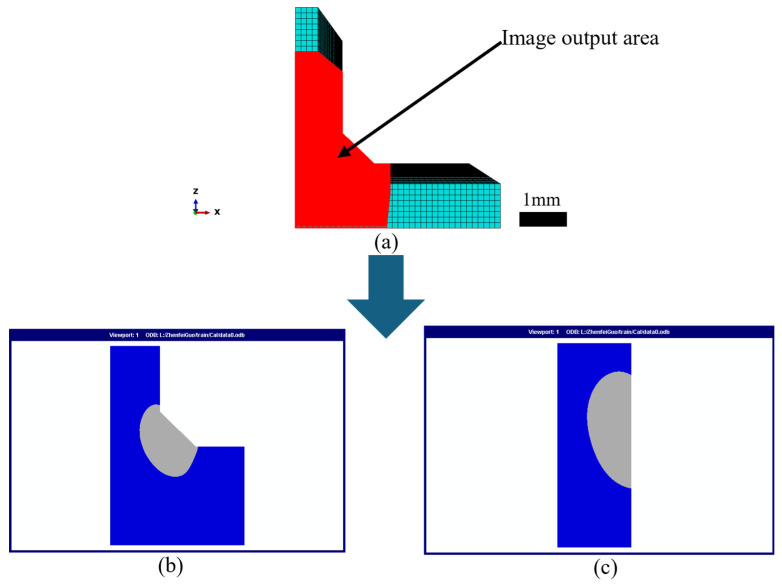
Representative weld morphology images extracted from FEM simulations: (**a**) output region, (**b**) parallel view, and (**c**) cross-sectional view.

After obtaining the nodal temperatures throughout the welding process by performing a transient thermal analysis, the weld morphology was extracted in the post-processing stage. During welding, the temperature field remains in a quasi-steady state for most of the time, and the shape of the molten pool remains nearly unchanged, except during arc initiation and termination where edge effects lead to some variations in the molten pool geometry [29]. Therefore, the weld cross-section perpendicular to the welding direction and located at the midpoint was recorded during post-processing as the representative weld morphology for the given heat source parameters. Moreover, to highlight weld details, only the region enclosed by the red rectangular box in Figure 3a was preserved for analysis. The molten pool zone was defined as the region with temperatures above 650 °C, consistent with the typical melting range of aluminum alloys (630–660 °C) [30]. Two weld morphology images—one perpendicular and one parallel to the welding direction—were extracted, as shown in Figure 3b,c.

#### 3.1.2. Heat Source Model

This study employs the Gaussian-cone composite heat source model in the finite element model to represent the total heat input (*q*_com_), thereby more accurately describing the thermal distribution characteristics of laser welding. The use of the Gaussian-cone composite model is primarily motivated by the model’s ability to overcome the limitations of a single heat source model and its better alignment with the actual physical processes involved in welding. A single Gaussian surface heat source or purely volumetric heat source can only generate simple weld seam shapes, failing to capture complex heat distribution patterns and accurately reproduce key geometric features such as the penetration depth and fusion zone, leading to significant deviations from the actual results. The Gaussian-cone composite model integrates the advantages of two types of heat sources. The Gaussian surface heat source (*q*_g_) corresponds to the surface heat input characteristics caused by plasma plume radiation, while the conical volumetric heat source (*q*_c_) represents the volume heat decay effect caused by back-cavity radiation absorption in the small hole. The synergistic effect of these two sources not only matches the energy transfer mechanisms of laser welding but also, through parameter adjustments, can reproduce various typical weld seam morphologies, fully covering the required weld seam geometric features for this study and providing a reliable morphological characterization foundation for subsequent heat source parameter identification.

**Figure 4:**
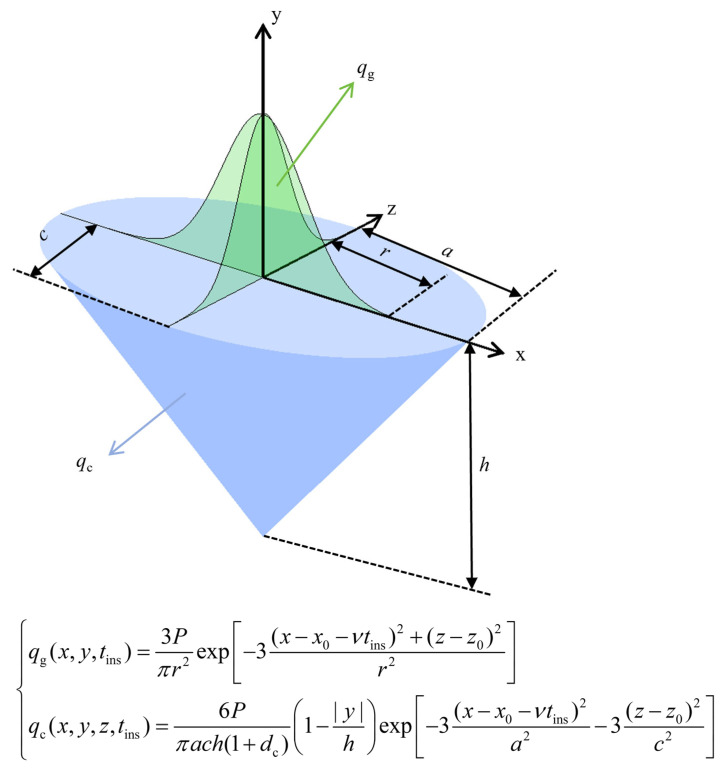
Schematic diagram of the heat source model.

Previous studies [31,32] have also adopted the Gaussian-cone composite heat source model, and practical results show that it performs excellently in laser welding simulations. Not only can it consistently reproduce thermal distribution during the welding process, but it also ensures the simulation accuracy of key weld geometric parameters, fully validating the model’s practicality and reliability, which further supports the choice of model in this study. The mathematical formulation of the combined heat source is given in Equation (11), and a schematic diagram is illustrated in Figure 4:

(11) $$q_{\mathrm{com}}(x,y,z,t_{\mathrm{ins}})=\beta q_{c}(x,y,z,t_{\mathrm{ins}})+(1-\beta )q_{g}(x,y,z,t_{\mathrm{ins}})$$

where *β* denotes the proportion of heat input from the Gaussian surface source, *t*_ins_ is the instantaneous time, and (*x*, *y*, *z*) denotes the coordinate of the weld.

During the laser welding process, a plasma plume with extremely high temperature forms above the molten pool is continuously emitting energy toward its surface [33]. This energy contribution is represented by a Gaussian surface heat source, which predominantly controls the weld width:

(12) $$q_{g}(x,y,t_{\mathrm{ins}})=\frac{3P}{\pi r^{2}}\exp[-3\frac{{\left(x-x_{0}-vt_{\mathrm{ins}}\right)}^{2}+{\left(z-z_{0}\right)}^{2}}{r^{2}}]$$

where *P* is the laser power, *q*_g_ is the heat flux generated by the Gaussian surface heat source model, *v* is the welding speed, *r* is the shape parameter of the Gaussian heat source model, and (*x*_0_, *y*_0_, *z*_0_) denotes the starting coordinate of the weld.

Due to the inverse Bremsstrahlung absorption inside the keyhole, laser energy is attenuated along the workpiece thickness [34]. Accordingly, a conical volumetric heat source model with linear attenuation through the thickness is adopted to govern weld penetration depth, and its mathematical formulation is given in [35]:

(13) $$q_{c}(x,y,z,t_{\mathrm{ins}})=\frac{6P}{\pi ach(1+d_{c})}\left(1-\frac{\left|y\right|}{h}\right)\exp\left[-3\frac{{\left(x-x_{0}-vt_{\mathrm{ins}}\right)}^{2}}{a^{2}}-3\frac{{\left(z-z_{0}\right)}^{2}}{c^{2}}\right]$$

where *q_c_* is the heat flux generated by the conical heat source model, *d_c_* is the heat source parameter affecting energy loss in the conical model, which is not considered in this study, *a* and *c* are the shape parameters of the conical heat source model, and *h* is the conical depth.

### 3.2. Analysis and Validation of FEM Simulation Results

**Figure 5:**
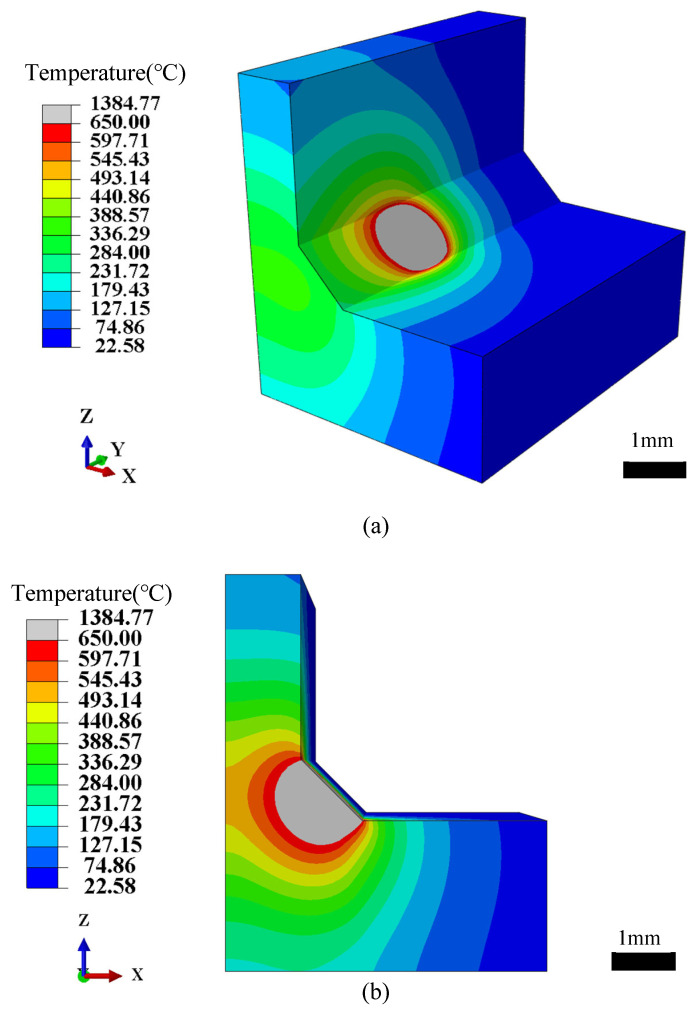
Simulated temperature field distributions: (**a**) three-dimensional view, (**b**) cross-sectional view.

Figure 5 presents the simulated temperature field of the laser wire-filling welding process, where the weld metal is mainly formed by melting 1.2 mm ER4047 aluminum alloy filler wire, accompanied by the partial melting of 2060-T3 and 2099-T3 Al–Li base materials, both contributing to the final weld metal.

Figure 5a illustrates the simulated temperature field contour of the double-sided T-joint under specific heat source parameters. A concentrated high-temperature zone is formed at the center of the heat-affected region, displaying a Gaussian-like distribution. Heat propagates three-dimensionally from the weld center toward the surrounding material, and the pronounced temperature gradients in the contour indicate intense thermal conduction, consistent with the localized high energy density input characteristic of laser welding. Furthermore, Figure 5b presents the cross-sectional temperature distribution, providing a clearer depiction of the temperature gradients and diffusion behavior within the molten pool and heat-affected zone.

From the overall distribution, the temperature field near the heat source exhibits a symmetrical pattern, which verifies the rationality of the assumed geometric and boundary condition symmetries in the model. This symmetry further confirms the accuracy of the combined heat source model in which the Gaussian surface source dominates the heat input at the weld surface, while the conical volumetric source accounts for the attenuation of laser energy through the thickness. A distinct attenuation trend in temperature is also observed along the direction perpendicular to the weld (i.e., through the plate thickness), with the temperature decreasing progressively from the central high-temperature zone to the surrounding low-temperature zone, thus highlighting a steep thermal gradient that governs the resulting weld geometry and microstructure. The temperature in the region surrounding the weld is significantly lower than that of the molten pool, indicating the presence of a narrow HAZ.

The weld cross-sectional profile was extracted from the simulation results and employed for comparative analysis with experimental observations, thereby providing crucial reference data for the subsequent construction of the HSPINet model. In summary, the simulated temperature field not only verifies the rationality of the adopted heat source model but also establishes a solid theoretical foundation for welding process control and weld quality optimization.

### 3.3. Sample Generation

**Figure 6:**
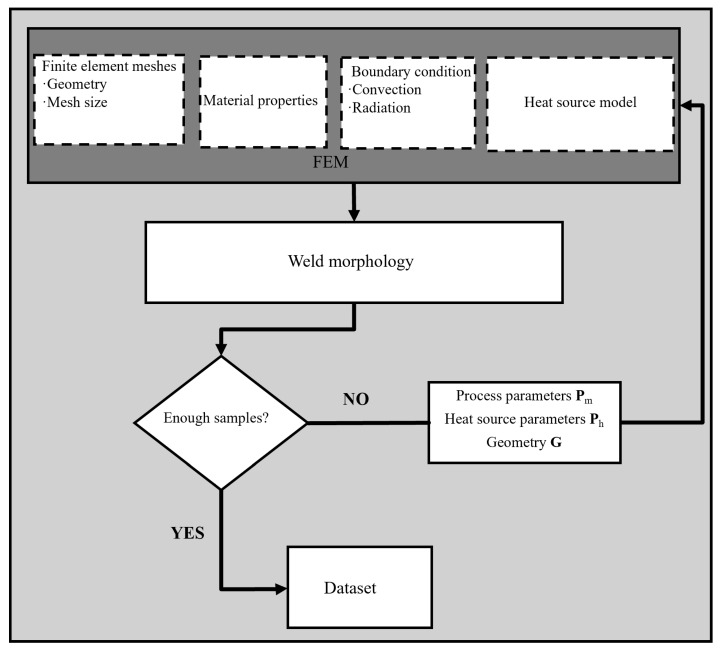
Flowchart of the dataset generation system.

As mentioned above, the heat source parameters to be identified in the combined heat source model are *r* and *h*, where *r* varies in the range of 0.2–3 and *h* in the range of 0.2–2. The parameter ranges were selected to prevent unphysical simulations—such as an excessively concentrated or overly dispersed heat input—while ensuring coverage of typical process conditions encountered in engineering practice. This strategy enhances the efficiency of sample generation and ensures that the dataset remains representative of realistic welding scenarios. The dataset generation process is illustrated in Figure 6, and the specific steps are described as follows.

A 3D thermal finite element model was established using Abaqus (2022), which incorporated temperature-dependent material properties together with convection and radiation boundary conditions. Heat source parameters were randomly sampled within the predefined ranges, and a Python(3.8.1)-based script was employed to generate a DFLUX subroutine corresponding to each parameter set, thereby defining both the spatial distribution and magnitude of the heat input. Subsequently, the temperature field over the entire welding process was computed for the current heat source parameters, and the results were subjected to post-processing. In the output images, gray represents weld regions with temperatures above the melting point, blue indicates non-weld areas, and white denotes the background. The processed weld images were saved, and the associated heat source parameters, **P**_h_, were recorded. Finally, a Python script was developed to automate the above procedure, invoking Abaqus for computation and post-processing in a loop. The complete set of weld images was consolidated to construct the dataset. A total of 50,000 training samples were generated using Latin hypercube sampling within the defined parameter ranges, and 2000 validation samples were randomly generated within the same range. A sample of the finite element model input parameters is shown in Table 2.

## 4. Development of the HSPINet Model

### 4.1. Residual Convolutional Neural Network

Convolutional neural networks (CNNs), inspired by the structure of biological visual systems, can automatically learn and extract features from images and have been extensively applied in tasks such as image classification, object detection, and semantic segmentation. Meanwhile, the inherent denoising capability of CNNs enables robust feature extraction even in the presence of blurred edges, background interference, and imaging noise [36]. In our previous work, this approach was validated using real weld cross-sectional images, yielding promising results [23].

Therefore, in this study, CNNs were employed to automatically extract weld morphology features associated with heat source parameters. The core component of a CNN is the convolutional layer, which extracts features of the weld fusion line by sliding convolutional kernels across the weld morphology images. By stacking multiple convolutional layers, a CNN can progressively learn more abstract and complex features. The convolutional layer operation can be expressed by Equation (14):

(14) $$Y_{i,j}^{l+l}=f\left(\underset{m,n}{\sum }X_{i+m,j+n}^{l}\cdot K_{m,n}+b_{i,j}\right)$$

where *Y* denotes the weld morphology features extracted by the current convolutional layer, *X* represents the weld morphology features input from the previous layer, *K* denotes the convolution kernel, *b* is the bias, and *f* is the activation function.

Pooling layers are typically added after convolutional layers to reduce the size of the weld morphology feature maps, thereby reducing the number of trainable parameters and computational cost, as well as enhancing the model’s invariance to local positional variations. Common pooling operations include max pooling and average pooling. In this study, max pooling with a window size of *p* × *p* was adopted to highlight the contribution of critical fusion-line features, and its operation is mathematically expressed as follows:

(15) $$P_{i,j}=\underset{0\leq m<p,0\leq n<p}{\max}X_{\left(i\cdot p+m,j\cdot p+n\right)}$$

Finally, the extracted weld fusion line features are concatenated with the process parameters and joint dimensions and subsequently mapped to the heat source parameter space through fully connected layers. This design embeds physical constraints into the mapping and, in combination with the multidimensional input, effectively mitigates the risk of non-unique solutions in the inverse problem. The mapping process can be mathematically expressed as follows:

(16) $$P_{h}=f(W\cdot (X⊕Z)+b)$$

where **P**_h_ denotes the heat source parameters predicted by the convolutional neural network, **W** represents the weight matrix, **Z** is a vector composed of process parameters and joint dimensions, and ⊕ denotes the stacking operation.

Before processing the data, we applied data normalization in order to accelerate the training process and improve model stability. Normalization adjusts the scale of the data so that it has zero mean and unit variances, which facilitates faster convergence of optimization algorithms [37]. The normalization formula can be expressed as

(17) $$X_{\mathrm{norm}}=\frac{X_{0}-\mu }{\sigma }$$

After the model is trained and used for prediction, in order to correlate the predicted results with their actual physical meanings, the predictions need to be denormalized, converting the normalized data back to its original scale so that the predicted results can intuitively reflect the actual heat source parameter values. The denormalization formula can be expressed as

(18) $$\hat{X}=X_{\mathrm{norm}}\cdot \sigma +\mu$$

In the above two equations, *X*_0_ is the original data, $\hat{X}$ is the denormalized data, *X*_norm_ is the normalized data, *σ* is the standard deviation of the data, and *μ* is the mean of the data.

During training, the Mean Squared Error (*MSE*) was adopted as the loss function to quantify the discrepancy between the predicted values and the ground truth, thereby guiding the updating and optimization of model parameters. The mathematical expression is given as follows:

(19) $$MSELoss=\frac{1}{N}\sum_{i=1}^{N}{\left(Y_{i}-{\hat{Y}}_{i}\right)}^{2}$$

where *MSELoss* is the loss value, *N* is the total number of samples, *Y_i_* is the ground truth of the *i*-th sample, and ${\hat{Y}}_{i}$ is the predicted value of the *i*-th sample. As a comprehensive metric reflecting the prediction accuracy of the model, minimizing the Mean Squared Error during training is a primary objective of this study.

During the model evaluation phase, in addition to using Mean Squared Error (*MSE*), the coefficient of determination (*R*^2^) and Relative Percentage Error (*RPE*) were also adopted to comprehensively assess the model’s predictive performance. These metrics help in understanding the model’s strengths and limitations from multiple perspectives and provide a basis for further model optimization. *R*^2^ is a key metric that measures the degree of fit between the predicted and actual values, and its calculation formula is as follows:

(20) $$R^{2}=1-\frac{\Sigma_{i=1}^{N}{\left(Y_{i}-{\hat{Y}}_{i}\right)}^{2}}{\Sigma_{i=1}^{N}{\left(Y_{i}-{\bar{Y}}_{i}\right)}^{2}}$$

where $\bar{Y_{i}}$ is the mean of the true values, and the other variables are as previously defined. The value of *R^2^* ranges from 0 to 1, where a value closer to 1 indicates a better fit of the model to the data and a smaller discrepancy between predicted and actual values; conversely, a value closer to 0 suggests a poor fit of the model. Driving the *R^2^* value as close to 1 as possible during training is the second major objective of this study.

The *RPE* is used to measure the relative deviation between predicted and actual values and can be expressed as follows:

(21) $$RPE=\frac{1}{N}\Sigma_{i=1}^{N}\left|\frac{Y_{i}-{\hat{Y}}_{i}}{Y_{i}}\right|\cdot 100\%$$

A smaller *RPE* value indicates better prediction accuracy of the model. It provides an intuitive measure of the relative difference between predicted and actual values. Minimizing the *RPE* value during training is the third key objective of this study.

As the number of network layers increases, the training error initially decreases but eventually saturates, and increasing the depth further may even cause it to rise. To address this problem, He et al. proposed the residual network (ResNet) based on the concept of identity mapping [38]. The core idea of a residual network lies in the residual block, which allows each newly added layer to more easily learn the identity function, thereby mitigating performance degradation:

(22) $$Y^{l+1}=h(X^{l})+F(X^{l},W^{l})$$

where *l* denotes the layer index, *Y*^*l*+1^ is the output of the (*l* + 1)-th layer, and *F*(*X^l^*,*W^l^*) represents the residual function (such as convolution, activation, etc.), which is used to learn the residual between the input *X^l^* and the output. *h*(*X^l^*) is an identity mapping, which directly adds the input to the output of the residual function to form the input for the next layer.

The choice of ResNet in this work was motivated by both data characteristics and physical relevance. Weld morphology images exhibit strong local spatial correlations and hierarchical features, which are effectively captured by convolutional layers combined with residual connections. Compared with alternative models, Vision Transformers [39] typically require very large datasets and are less efficient in extracting fine-grained details, while Graph Neural Networks [40] are designed for non-Euclidean data structures rather than grid-based image data. Simpler models such as MLPs or tree-based approaches [41] either risk overfitting due to excessive parameters or fail to capture the nonlinear spatial patterns inherent in weld images. ResNet therefore provides a practical balance, offering both high predictive accuracy and computational efficiency, while remaining consistent with the localized physical mechanisms of welding.

In the CNN model proposed in this study, residual blocks are embedded between convolutional layers, particularly in the deeper layers. This design allows the network to efficiently capture high-level weld features while alleviating the vanishing gradient problem, thereby improving both the generalization ability and predictive accuracy.

To further enhance the model’s capability in capturing critical features within weld morphology images, an attention mechanism is introduced in this study. The core concept of the attention mechanism is inspired by human visual attention, which allows the model to dynamically emphasize the most informative regions of the input data, thereby improving both accuracy and computational efficiency. In the task of identifying welding heat source parameters, certain regions in weld morphology images—such as the fusion line and HAZ—are particularly influential for parameter prediction. However, these regions often occupy only a small proportion of the overall image and are easily overwhelmed by redundant background information. Without attention, a CNN may fail to sufficiently emphasize such sparse yet critical features, resulting in reduced prediction accuracy, particularly when multiple process parameters and varying plate thicknesses are involved. By integrating the attention mechanism, the model can automatically learn the importance weights of these key regions, recalibrate feature responses, and suppress irrelevant information, thereby enabling more effective feature extraction and ensuring a stable performance across diverse welding conditions.

The spatial attention mechanism dynamically adjusts the weights of different spatial locations in the feature map by learning a spatial attention map from the input feature representation, thereby enabling the model to focus more effectively on the most informative regions [42]. Unlike the self-attention mechanism in Transformer architectures, which models pairwise dependencies among sequential tokens, the spatial attention used here provides efficient local feature enhancement at a lower computational cost while still capturing the essential physical patterns of weld morphology. For future work, Transformer-style self-attention mechanisms may be explored to further model long-range dependencies in weld morphology features.

The spatial attention mechanism is implemented as follows:

(1) Average pooling and max pooling of the feature map. The input feature map is processed by average pooling and max pooling along the channel dimension, producing two one-dimensional vectors. These vectors encode the mean and maximum responses across channels, thereby capturing complementary global information from the feature map.
(2) Activation and application of the attention map. The two vectors produced by average and max pooling are concatenated and subsequently passed through a convolutional layer to achieve feature fusion, generating an attention map. Each element of this map indicates the relative importance of the corresponding spatial location in the feature map. In the model proposed in this study, the spatial attention mechanism is embedded between the convolutional and pooling layers. By introducing the spatial attention mechanism, the model can better capture key features in weld morphology images, thereby improving the prediction accuracy of heat source parameters.

### 4.2. Model Training and Validation

The CNN model employed in this study is designed for fused feature extraction based on images and process parameters, as well as for heat source parameter prediction. The architectural design of the model effectively captures the weld morphology features embedded in the images. By integrating process information, the model enhances the regression performance for predicting heat source parameters. The overall workflow primarily includes four aspects: data preprocessing, model architecture design, training configuration, and evaluation metrics.

In terms of data preprocessing, each sample consists of two weld images (captured from different angles or positions) together with a set of numerical parameters, including two process parameters, one joint dimension, and four heat source parameters. For an input image, a smaller height and width result in a smaller output feature map, thereby reducing the computational cost of convolution operations. To balance efficiency and accuracy, the original 736 × 496 images were converted to grayscale and uniformly resized to 59 × 143, which not only reduces computational load and accelerates training but also retains the essential weld morphology features. This resolution was determined through multiple down-sampling trials as an optimal balance between accuracy and efficiency. The two images in the same sample group are concatenated to form a two-channel input for the CNN. Additionally, both the process parameters and the target heat source parameters were normalized using the precomputed mean and standard deviation, ensuring stable and efficient training.

In terms of model architecture, the CNN consists of five consecutive convolution-pooling modules. For a convolution kernel of size *F_H_* × *F_D_*, when applied to an input image of size *H*_1_ × *H*_2_, with padding *λ* and stride *s*, the size of the output feature map is calculated as follows:

(23) $$H\cdot D=\left(\frac{H_{1}-F_{H}+2\lambda }{s}+1\right)\left(\frac{D_{1}-F_{D}+2\lambda }{s}+1\right)$$

where *H* and *D* denote the height and width of the output feature map, respectively.

**Figure 7:**
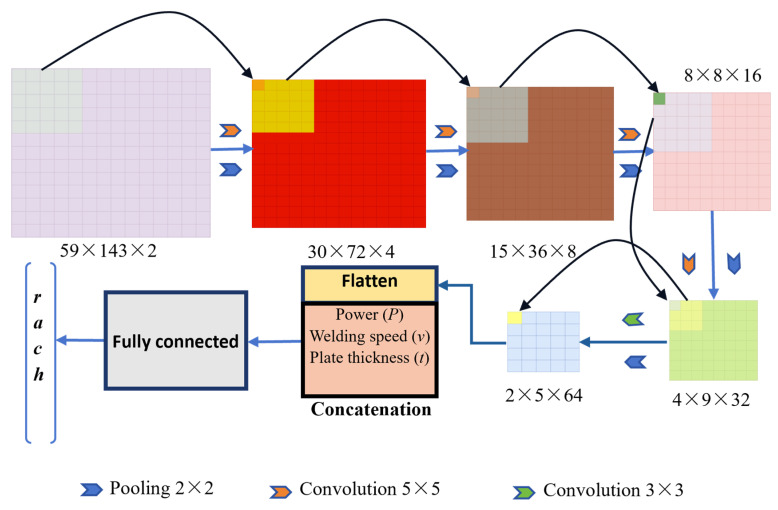
Architecture of the HSPINet model.

During the five convolution operations, the kernel size is 5 × 5, with a stride of 1 and padding of 2. According to Equation (23), this setting ensures that the image size remains unchanged. In the subsequent five max pooling operations following each convolution, the output feature size is computed using Equation (15), with results rounded up. The pooling kernel size is 2 × 2 with a stride of 2. To guarantee that both the height and width of the feature map are halved, the padding is set to 1 when the input dimensions are odd and 0 when even. Each module progressively increases the number of channels, with multiple convolution kernels extracting diverse features from the image after each convolution. Starting from two channels, the model expands through five stages (4, 8, 16, 32, and 64), gradually compressing the spatial dimensions. An ReLU activation function is applied after each convolutional layer to prevent vanishing gradients and to stabilize training. After extracting the final convolutional features, they are flattened into a one-dimensional vector and concatenated with the normalized process parameters. The combined vector is then fed into a fully connected network for prediction. The fully connected part includes two hidden layers with dimensions of 1024 and 1500, respectively. A dropout function is applied after the fully connected layers to mitigate the risk of overfitting. The output layer has a dimensionality of four, corresponding to the four heat source parameters (*r*, *a*, *c*, and *h*). Furthermore, diverse process parameters such as laser power, welding speed, and joint thickness were incorporated during dataset generation to improve the model’s generalization capability under different welding conditions, as shown in Figure 7. The structural parameters of the operations in Figure 7 refer to Table 3.

In terms of training configuration, the Adam optimizer is adopted, which is an optimization algorithm based on the adaptive estimation of gradients. It combines momentum and adaptive learning rate adjustment mechanisms to automatically adjust the learning rate based on the history of parameter updates, thereby providing a more efficient optimization path during training. The learning rate is set to 1 × 10^−4^, which allows for fine-grained parameter updates in the early training stages, helping to prevent gradient explosion or unstable convergence, while in the later stages it facilitates a closer convergence to the global or local optimum, ultimately enhancing performance. The loss function is *MSELoss*, as previously described. The total number of training epochs is set to 100, and the batch size is set to 32. A larger batch size provides more accurate gradient estimates, aiding in more stable convergence. On the other hand, a smaller batch size introduces a certain level of noise, which can help the model escape local optima and improve its generalization capability. Therefore, this study adopts a batch size of 32 as a compromise to achieve optimal model performance. During training, loss curves on both the training and validation sets are recorded to monitor the convergence process and generalization performance. The model with the best performance is ultimately retained for evaluation. The hyperparameters are fine-tuned by comparing the *RPE* of different configurations on the validation set, which ensured a balance between model accuracy and generalization while avoiding overfitting.

Two key evaluation metrics are employed. The coefficient of determination (*R*^2^), which quantifies the agreement between predicted and actual values, and the *RPE*, which measures the percentage of relative error. In addition, a visualization analysis is conducted for *MSELoss* and the prediction results of each heat source parameter. In the visualization of heat source parameters, comparison curves between predicted and actual values are plotted, providing an intuitive display of the model’s fitting capability for different target parameters.

**Figure 8:**
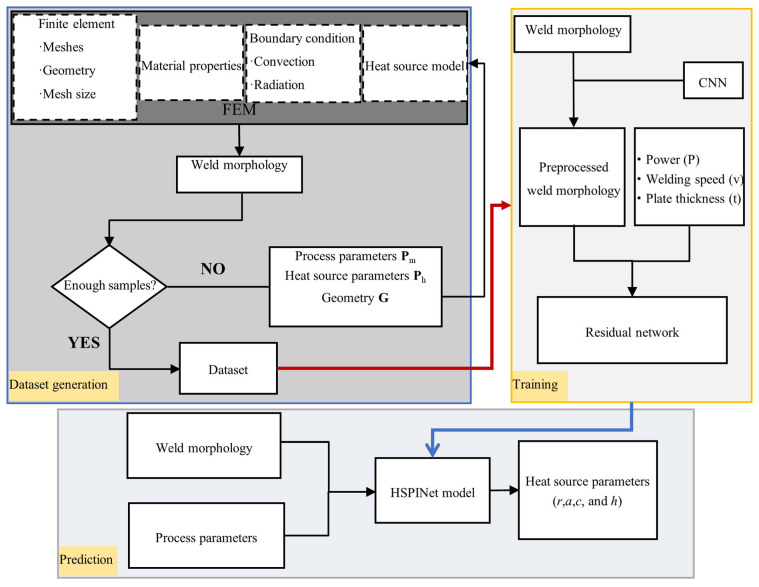
End-to-end workflow of the HSPINet framework.

The overall workflow of the proposed method is illustrated in Figure 8, which presents an end-to-end schematic of the inversion framework. It systematically outlines the dataset generation through finite element simulations, the deep learning model training using multi-view weld morphology and process parameters, and the final prediction of heat source parameters.

In summary, the proposed model leverages the synergy between image features and process parameters, thereby demonstrating the potential of multimodal input for identifying welding heat source parameters. It also ensures reproducibility and interpretability through carefully designed training and evaluation mechanisms. Moreover, the overall workflow is not restricted to T-joints but can be readily extended to other welding structures, such as lap joints, with only minor adjustments in the dataset generation stage to reflect the corresponding conditions. Although this paper does not present experiments on other joint types, we have systematically validated the effectiveness of the proposed method in butt joints in our prior work [23], which further supports the generalizability and applicability of the approach.

### 4.3. Results and Discussion

**Figure 9:**
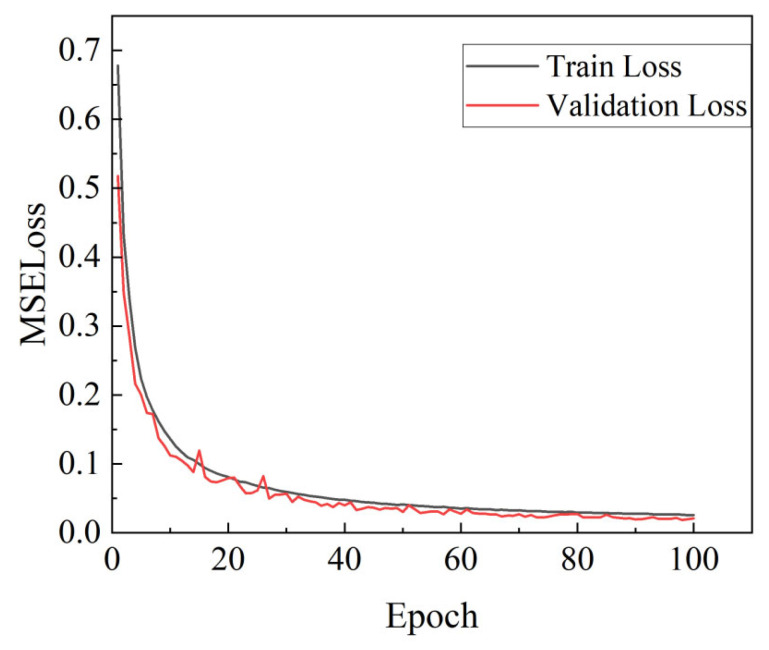
Trend of *MSELoss* with epoch variation.

During the model training process, the first objective of this study—the gradual decrease in the training set *MSELoss*—was achieved, dropping from 0.69 in the first epoch to 0.03 in the 100th epoch. Although the validation set loss fluctuated, it exhibited an overall decreasing trend. The average loss error for the validation set was 0.02, as shown in Figure 9.

As illustrated in Figure 9, the model effectively optimized its weight during the training process, leading to a gradual reduction in prediction error. In the early stages of training, both the training loss (blue curve) and validation loss (orange curve) exhibit a downward trend, indicating that the model is able to quickly adapt to the data, and it learns effectively. As the number of training epochs increases, the loss curves gradually flatten and the rate of decline decreases, suggesting that the model is approaching convergence, and the optimization effect in the later training stage diminishes. Observing the curves reveals that the validation loss shows some fluctuation at the beginning of training, but it gradually stabilizes and eventually becomes close to the training loss, indicating a good performance on the training set and strong generalization ability, enabling accurate predictions on unseen data.

For the prediction results of each heat source parameter, the second evaluation objective of this study is assessed using the coefficient of determination (*R*^2^), which serves as an effective analytical metric, as summarized in Table 4.

The results, with *R*^2^ values all exceeding 0.95, demonstrate that the model exhibits a high predictive performance for all four heat source parameters. The high *R*^2^ values benefit from the rich spatial information contained in weld morphology images and the use of process parameters as auxiliary inputs, which together constrain the solution space. Combined with the large and diverse training dataset, these factors enable the model to achieve a stable convergence and high prediction accuracy. In particular, the *R*^2^ value approaching 1 for the parameter *h* indicates that the model can predict this parameter with very high accuracy. This may be attributed to the fact that the *h* parameter is typically related to the thickness of the welded workpiece, which is a stable and easily measurable physical quantity in actual welding processes. Such stability results in low noise and the strong regularity of this parameter in the training data, which facilitates the model in learning its mapping relationship with the input variables. In addition, the range of variation for the conical height is relatively narrow, further enhancing the model’s ability to fit this parameter. Compared to other heat source parameters that are more strongly influenced by multiple coupled factors, the prediction results for conical height generally exhibit greater accuracy and stability.

In contrast, the *R*^2^ value of 0.96 for the heat source radius, *r*, is slightly lower than that of the other parameters. The radius determines the spatial distribution range of the heat input and is simultaneously affected by multiple factors, including welding power, scanning speed, material thermal conductivity, and heat source offset. These influences are often nonlinearly coupled and subject to uncertainty during the welding process, which increases the complexity of learning this parameter. Compared with the conical height *h*, which is relatively concentrated and regular, the value range of *r* in the dataset is more dispersed, leading to greater sample variability during model fitting. If the thermal field features corresponding to different heat source radii are not sufficiently distinct or show a high similarity, the model may struggle to effectively discriminate them, thereby reducing prediction accuracy. In addition, the effect of the heat source radius on thermal field morphology is relatively localized, meaning its impact on the overall temperature distribution is weaker than that of the other parameters. Consequently, its contribution to the loss function becomes attenuated by more dominant features, making it difficult for the model to fully capture its variation patterns.

The third objective of this study—the *RPE*—was achieved at 6.38%, as shown in Table 4. This indicates that the model has a relatively small prediction error and high overall accuracy. Although the *RPE* in the range of 5% to 10% is acceptable, it does not yet represent an extremely low error level, suggesting that the model still exhibits certain deviations on some samples. This phenomenon may be caused by local blur or unclear textures in the input data that introduce interfering features or by overly large or small outliers in the process parameters and joint size concatenated with the image features, which affect the accuracy of the model’s feature extraction. It could also be due to distributional differences between the training and testing sets, leading to a slightly insufficient generalization ability under boundary samples or complex conditions. The current model structure shows a limited representational capacity for multi-parameter regression, particularly in capturing the mapping between fine-grained image features and continuous variables, indicating the scope for further improvement.

**Figure 10:**
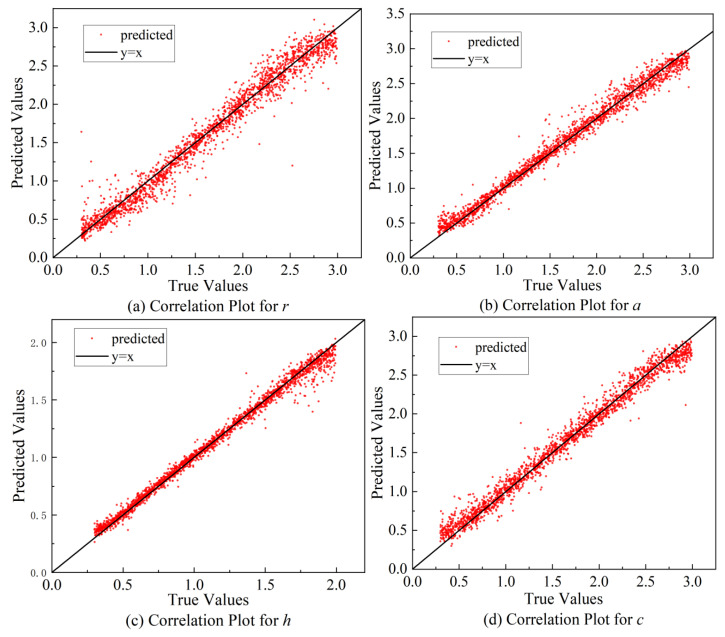
Scatter plots comparing predicted and true values of parameters.

The HSPINet model proposed in this study also outputs comparison plots between the predicted and actual values of the four target heat source parameters, as shown in Figure 10.

The test set used in this study comprises 2000 data samples, corresponding to 2000 scatter points in the comparison plot. The horizontal and vertical axes represent the value range of each heat source parameter, where the horizontal axis denotes the true values and the vertical axis denotes the predicted values. A y = x reference line is added to the plot to facilitate the comparison of the scatter point distribution. Figure 10 shows that the majority of scatter points are closely distributed around the reference line, indicating that the differences between predicted and true values are minimal with only a few points significantly above or below the line.

The predicted values exhibit a strong overall consistency with the ground truth, reflecting the model’s robust global learning capability and its effectiveness in capturing inter-sample parameter variation patterns. The scatter points for parameter *h* exhibit the closest fit, with the predicted and true value curves nearly overlapping, suggesting that the model demonstrates a strong modeling ability for this physically well-defined and narrowly distributed parameter. For parameters *a* and *c*, although the overall fitting trend is accurate, certain deviations are observed in individual samples, which may be due to weak corresponding information in the image features or the influence of multiple coupled factors affecting these parameters. The scatter points for parameter *r* show slight fluctuations in some local regions, which are likely due to the high variability of this parameter under different operating conditions, influenced by complex factors such as energy distribution and the scanning path, resulting in the model’s relatively limited ability to capture its local variations.

Regarding the accuracy and error of the prediction results, please refer to Table 5.

To further elucidate the physical meaning of feature extraction, we visualized the weld image features captured by each convolution–pooling layer. The corresponding visualization results are provided in the Supplementary Materials (Table S1). The results show that the shallow layers (L1–L2) primarily capture weld boundaries and fusion line contours, while deeper layers (L3–L5) progressively extract key morphological features such as bead width and penetration depth. These features are directly related to heat source parameters, indicating that the model’s predictions are grounded in welding physics rather than relying solely on data-driven fitting.

The HSPINet model demonstrates a strong predictive capability for heat source parameters in the laser welding process. By effectively handling multimodal data from composite heat source models, it can accurately capture parameter variations, with only slight performance differences among specific parameters. From an industrial perspective, the predicted heat source parameters serve as mathematical descriptors of the heat input distribution in finite element simulations. In practice, weld morphology images obtained from metallographic cross-sections can be directly input into the trained model to derive the corresponding heat source parameters, which can then be applied to high-fidelity FEM simulations for deformation and residual stress evaluation. This not only supports the optimization and reliability assessment of welded joints but also highlights the potential for integrating deep learning with physics-based modeling. Nevertheless, the robustness of the model is constrained by the predefined training parameter range; extrapolation beyond this range may introduce significant uncertainty due to the absence of learned physical patterns, in which case, FEM validation remains necessary. Future work will therefore focus on enhancing the extrapolation capability under extreme conditions through active learning, data augmentation, and physics-informed constraints.

The innovations of this study are mainly reflected in the following three aspects: first, a multi-view weld, morphology-constrained inversion method is proposed, which incorporates both cross-sectional and longitudinal weld images to enhance the stability and reliability of the inversion process. Second, a physics-embedded cross-modal mapping network is constructed, which integrates process parameters with morphological features, thereby effectively preventing the generation of physically inconsistent solutions. Finally, a generalized prediction framework with adaptability to both process variations and joint dimensions is established, significantly enhancing the engineering applicability and scalability of the method. Compared with traditional approaches such as response surface models (RSM), which often suffer from underfitting or overfitting, limited feature representation, and weak transferability across different joint types, the proposed HSPINet directly learns the nonlinear mapping from weld morphology and process parameters to heat source parameters in an end-to-end manner. It not only improves prediction accuracy and efficiency but also demonstrates a strong generalization to varying welding conditions, making it more practical for engineering applications.

## 5. Conclusions

In this study, an attention-enhanced residual convolutional neural network (HSPINet) is developed to identify composite heat source parameters in the laser welding of T-joint structures. The proposed approach is validated to be feasible and effective. The main conclusions are as follows:

(1) A high-fidelity dataset was constructed by performing the finite element thermal analyses under diverse process parameters and heat source conditions. The dataset effectively captured the relationship between weld pool geometries and heat source characteristics, providing a robust foundation for training the deep learning model.
(2) The proposed HSPINet successfully established mapping between multi-angle weld cross-sectional morphologies, process parameters, and the underlying composite heat source parameters. The model demonstrated an excellent predictive performance, achieving an *RPE* below 7% and an average coefficient of determination (*R*^2^) of 0.98 on the testing dataset, indicating strong prediction accuracy and generalization capability.
(3) The results confirm that the method can reliably identify heat source parameters across varying process settings and joint dimensions, significantly enhancing the efficiency and precision of heat source parameter identification. This work provides a valuable technical basis for improving the fidelity of welding thermal simulations and mechanical performance evaluations in complex engineering applications.

## Figures and Tables

**Table 1:** Convection coefficients of aluminum alloys at different temperatures.

Temperature (°C)	20	100	500	750	1000	1500	2000	2500	3000
Convection coefficient (W/m^2^·K)	1.366	6.680	33.400	91.150	148.900	264.400	379.900	580.000	800.000

**Table 2:** Sample space.

Input Parameters of the Finite Element Model		Range
Process Parameters	Power (W)	[2000, 3500]
	Welding Speed (m/min)	[3, 4.5]
	Thickness (mm)	[1, 3]
Heat Source Parameters	*r* (mm)	[0.3, 3]
	*a* (mm)	[0.3, 3]
	*c* (mm)	[0.3, 3]
	*h* (mm)	[0.3, 2]

**Table 3:** Structural parameters of the HSPINet model.

Name	Operation	Kernel Size	Stride	Quantity/Method	Padding	Activation Function
L1-C	Convolution	5 × 5	(1, 1)	Kernels. 4	padding = 2	ReLU
L1-P	Pooling	2 × 2	(2, 2)	Max Pooling	padding = (1, 1)	-
L2-C	Convolution	5 × 5	(1, 1)	Kernels. 8	padding = 2	ReLU
L2-P	Pooling	2 × 2	(2, 2)	Max Pooling	padding = (0, 0)	-
L3-C	Convolution	5 × 5	(1, 1)	Kernels. 16	padding = 2	ReLU
L3-P	Pooling	2 × 2	(2, 2)	Max Pooling	padding = (1, 0)	-
L4-C	Convolution	5 × 5	(1, 1)	Kernels. 32	padding = 2	ReLU
L4-P	Pooling	2 × 2	(2, 2)	Max Pooling	padding = (0, 0)	-
L5-C	Convolution	3 × 3	(1, 1)	Kernels. 64	padding = 2	ReLU
L5-P	Pooling	2 × 2	(2, 2)	Max Pooling	padding = (0, 1)	-
L6-F	Fully Connected	-	-	Dimension. 1024	-	-
L7-F	Fully Connected	-	-	Dimension. 1500	-	-
L8-F	Fully Connected	-	-	Dimension. 4	-	-

**Table 4:** Performance evaluation metrics and results of the HSPINet model.

Evaluation Metric	Test Set Average Loss	*R*^2^ for *r*	*R*^2^ for *a*	*R*^2^ for *h*	*R*^2^ for *c*	Test Set *RPE*
Value	0.02	0.96	0.98	0.99	0.98	6.38%

**Table 5:** Comparison of model inputs, true values, and predicted outputs.

Parameter	Model Input	Actual Input Value/ Output Error	Predicted Result	Actual Output Value
Power (W)	3413.25	3413.25	-	-
Welding Speed (m/min)	3.79	3.79	-	-
Joint Thickness (mm)	1.84	1.84	-	-
*r* (mm)	-	2.55%	2.28	2.34
*a* (mm)	-	2.36%	1.16	1.13
*h* (mm)	-	2.23%	1.25	1.28
*c* (mm)	-	1.01%	1.33	1.32

## Data Availability

The data that support the findings of this study are available from the corresponding author upon reasonable request.

## Supplementary Materials

The following supporting information can be downloaded at: https://www.mdpi.com/article/10.3390/ma18174174/s1, Table S1: Visualization of weld image features extracted across CNN layers of HSPINet.

## Abbreviations

The following abbreviations are used in this manuscript.

CWM	Computational Welding Mechanics
FEM	Finite Element Method
CNN	Convolutional Neural Network
ResNet	Residual Network
HSPINet	Heat Source Parameter Identification Network
*RPE*	Relative Percentage Error
*MSE*	Mean Squared Error
*R*^2^	Coefficient of Determination
RSM	Response Surface Model
LBW	Laser Beam Welding
GTAW	Gas Tungsten Arc Welding
HAZ	Heat-Affected Zone

## References

[1] Chen Q., Zhang L., Chen M.-T., Zhao O. (2024). Experimental Investigation and Predictive Models of Membrane Residual Stresses in S690 High Strength Steel Welded π-Shaped and Cruciform Sections. Eng. Struct..

[2] Guo Z., Bai R., Lei Z., Jiang H., Zou J., Yan C. (2021). Experimental and Numerical Investigation on Ultimate Strength of Laser-Welded Stiffened Plates Considering Welding Deformation and Residual Stresses. Ocean Eng..

[3] Bai R., Guo Z., Lei Z., Wu W., Yan C. (2019). Hybrid Inversion Method and Sensitivity Analysis of Inherent Deformations of Welded Joints. Adv. Eng. Softw..

[4] Liu Z., Jin X., Li J., Hao Z., Zhang J. (2022). Numerical Simulation and Experimental Analysis on the Deformation and Residual Stress in Trailing Ultrasonic Vibration Assisted Laser Welding. Adv. Eng. Softw..

[5] Xu J., Ma Y., Wang L., Lu X. (2022). Numerical Simulation of Arc and Metal Transfer Behaviors in Double-Wire Gas Metal Arc Welding. Weld. World.

[6] Farias R.M., Teixeira P.R.F., Vilarinho L.O. (2022). Variable Profile Heat Source Models for Numerical Simulations of Arc Welding Processes. Int. J. Therm. Sci..

[7] Yan H., Zeng X., Cui Y., Zou D. (2024). Numerical and Experimental Study of Residual Stress in Multi-Pass Laser Welded 5A06 Alloy Ultra-Thick Plate. J. Mater. Res. Technol..

[8] Sun J., Klassen J., Nitschke-Pagel T., Dilger K. (2018). Effects of Heat Source Geometric Parameters and Arc Efficiency on Welding Temperature Field, Residual Stress, and Distortion in Thin-Plate Full-Penetration Welds. Int. J. Adv. Manuf. Technol..

[9] Raftar H.R., Ahola A., Lipiäinen K., Björk T. (2023). Simulation and Experiment on Residual Stress and Deflection of Cruciform Welded Joints. J. Constr. Steel Res..

[10] Yan L., Guan Y., Luo K., Wang Q. (2023). Corrugated Steel Web I-Girder Welding Deformation and Residual Stress Research. Structures.

[11] Ghafouri M., Ahola A., Ahn J., Björk T. (2022). Numerical and Experimental Investigations on the Welding Residual Stresses and Distortions of the Short Fillet Welds in High Strength Steel Plates. Eng. Struct..

[12] Walker T.R., Bennett C.J. (2019). An Automated Inverse Method to Calibrate Thermal Finite Element Models for Numerical Welding Applications. J. Manuf. Process..

[13] Farias R.M., Teixeira P.R.F., Vilarinho L.O. (2021). An Efficient Computational Approach for Heat Source Optimization in Numerical Simulations of Arc Welding Processes. J. Constr. Steel Res..

[14] Jiao H., Jin H. (2024). An Automated Optimization Procedure for Geometry Parameters Calibration of Two-Curvature Conical Heat Source Model. Int. J. Therm. Sci..

[15] Bai R., Guo Z., Tian C., Lei Z., Yan C., Tao W. (2018). Study on Welding Sequence of Butt-Welded Structures Based on Equivalent Heat Source Parameter. Int. J. Press. Vessel. Pip..

[16] Bai R., Guo Z., Tian C., Lei Z., Yan C., Tao W. (2018). Investigation on Welding Sequence of I-Beam by Hybrid Inversion. Mar. Struct..

[17] Jiao H., Jin H. (2023). An Inverse Method for Determining Geometric Parameters of Heat Source Models Using Analytical Solutions and Regression Analysis. J. Mech. Sci. Technol..

[18] Guo Z., Jiang H., Bai R., Lei Z. (2024). Prediction of Laser-Welded Deformation Using Artificial Neural Networks. J. Laser Appl..

[19] Zhang Z., Wen G., Chen S. (2019). Weld Image Deep Learning-Based on-Line Defects Detection Using Convolutional Neural Networks for Al Alloy in Robotic Arc Welding. J. Manuf. Process..

[20] Fu G., Lourenço M.I., Duan M., Estefen S.F. (2016). Influence of the Welding Sequence on Residual Stress and Distortion of Fillet Welded Structures. Mar. Struct..

[21] Fu G., Gu J., Lourenco M.I., Duan M., Estefen S.F. (2015). Parameter Determination of Double-Ellipsoidal Heat Source Model and Its Application in the Multi-Pass Welding Process. Ships Offshore Struct..

[22] Kitano H., Mikami Y. (2022). Constructing a Heat Source Parameter Estimation Model for Heat Conduction Finite Element Analysis Using Deep Convolutional Neural Network. Mater. Today Commun..

[23] Guo Z., Jiang H., He L., Lei Z., Bai R. (2024). CNN-Empowered Identification of Heat Source Parameters from the Cross-Section Profile of Laser-Welded Zone. Int. J. Adv. Manuf. Technol..

[24] Tsirkas S.A. (2018). Numerical Simulation of the Laser Welding Process for the Prediction of Temperature Distribution on Welded Aluminium Aircraft Components. Opt. Laser Technol..

[25] Brown S., Song H. (1992). Finite Element Simulation of Welding of Large Structures. J. Eng. Ind..

[26] Russo Spena P., De Maddis M., Razza V., Santoro L., Mamarayimov H., Basile D. (2025). Infrared-Guided Thermal Cycles in FEM Simulation of Laser Welding of Thin Aluminium Alloy Sheets. Metals.

[27] Li S., Ren S., Zhang Y., Deng D., Murakawa H. (2017). Numerical Investigation of Formation Mechanism of Welding Residual Stress in P92 Steel Multi-Pass Joints. J. Mater. Process. Technol..

[28] Zhao K., Wang B., Xue H., Wang Z. (2022). Influence of Material Randomness on Welding Residual Stress in Dissimilar Metal Welded Joints of Nuclear Power Plants. Int. J. Front. Eng. Technol..

[29] Guo W., Kar A. (2000). Determination of Weld Pool Shape and Temperature Distribution by Solving Three-Dimensional Phase Change Heat Conduction Problem. Sci. Technol. Weld. Join..

[30] Jin Y., Wang S., Huang W., Li L. (2019). Numerical Simulation Analysis and Experimental Verification of Laser Beam Welding of New Type Al-Li Alloy. Hot Work. Technol..

[31] Mwema F.M. (2017). Transient Thermal Modeling in Laser Welding of Metallic/Nonmetallic Joints Using SolidWorks^®^ Software. IJNM.

[32] Escribano-García R., Álvarez P., Marquez-Monje D. (2022). Calibration of Finite Element Model of Titanium Laser Welding by Fractional Factorial Design. JMMP.

[33] Yang Z., Tao W., Li L., Chen Y., Shi C. (2017). Numerical Simulation of Heat Transfer and Fluid Flow during Double-Sided Laser Beam Welding of T-Joints for Aluminum Aircraft Fuselage Panels. Opt. Laser Technol..

[34] Tao W., Yang Z., Shi C., Dong D. (2017). Simulating Effects of Welding Speed on Melt Flow and Porosity Formation during Double-Sided Laser Beam Welding of AA6056-T4/AA6156-T6 Aluminum Alloy T-Joint. J. Alloys Compd..

[35] Lundbäck A., Runnemalm H. (2005). Validation of Three-Dimensional Finite Element Model for Electron Beam Welding of Inconel 718. Sci. Technol. Weld. Join..

[36] Ilesanmi A.E., Ilesanmi T.O. (2021). Methods for Image Denoising Using Convolutional Neural Network: A Review. Complex Intell. Syst..

[37] Ioffe S., Szegedy C. (2015). Batch Normalization: Accelerating Deep Network Training by Reducing Internal Covariate Shift. arXiv.

[38] He K., Zhang X., Ren S., Sun J. (2016). Deep Residual Learning for Image Recognition. Proceedings of the 2016 IEEE Conference on Computer Vision and Pattern Recognition (CVPR).

[39] Whitman S.E., Latypov M.I. (2025). Machine Learning of Microstructure–Property Relationships in Materials Leveraging Microstructure Representation from Foundational Vision Transformers. Acta Mater..

[40] Fan Z., Ma E. (2021). Predicting Orientation-Dependent Plastic Susceptibility from Static Structure in Amorphous Solids via Deep Learning. Nat. Commun..

[41] Liu C., Wang Y., Wang Y., Islam M., Hwang J., Wang Y., Fan Y. (2023). Concurrent Prediction of Metallic Glasses’ Global Energy and Internal Structural Heterogeneity by Interpretable Machine Learning. Acta Mater..

[42] Woo S., Park J., Lee J.-Y., Kweon I.S., Ferrari V., Hebert M., Sminchisescu C., Weiss Y. (2018). CBAM: Convolutional Block Attention Module. Computer Vision–ECCV 2018.

