# Supplementary material for: Heat Source Parameter Identification Based on Attention-Enhanced Residual Convolutional Neural Network
Source: Materials (Basel). 2025 Sep 5;18(17):4174. doi: 10.3390/ma18174174 (PMC12430179; doi:10.3390/ma18174174)
Supplement: Supplementary file 1 [file materials-18-04174-s001.zip › materials-3791240-supplementary.pdf]

Table S1 Visualization of weld image features extracted across CNN layers of HSPINet

**Layer index**	**Size**	**Image**
Input layer	59×14 3	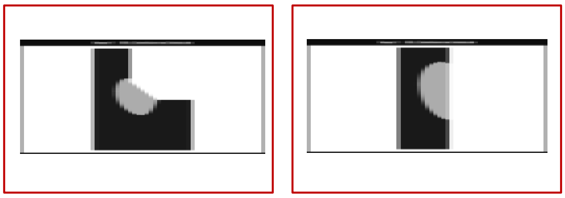
L1	30×72	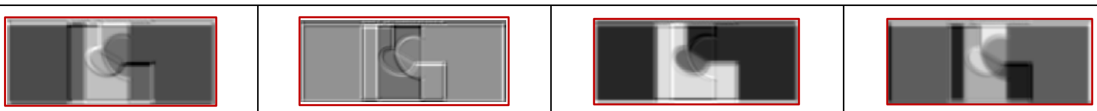
L2	15×36	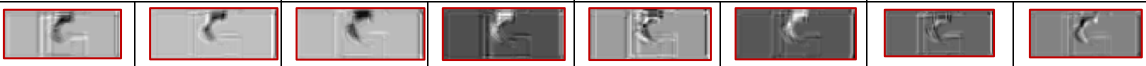
L3	8×8	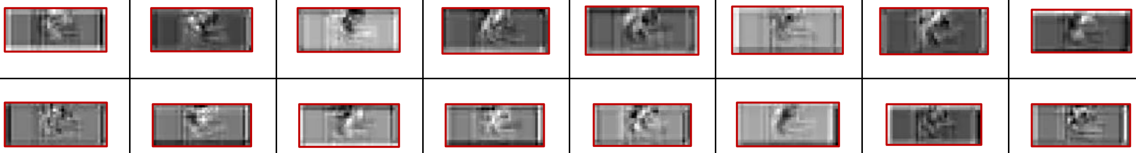
L4	4×9	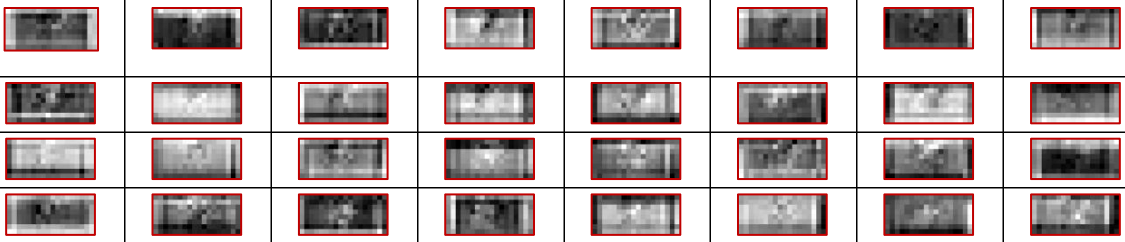
L5	2×5	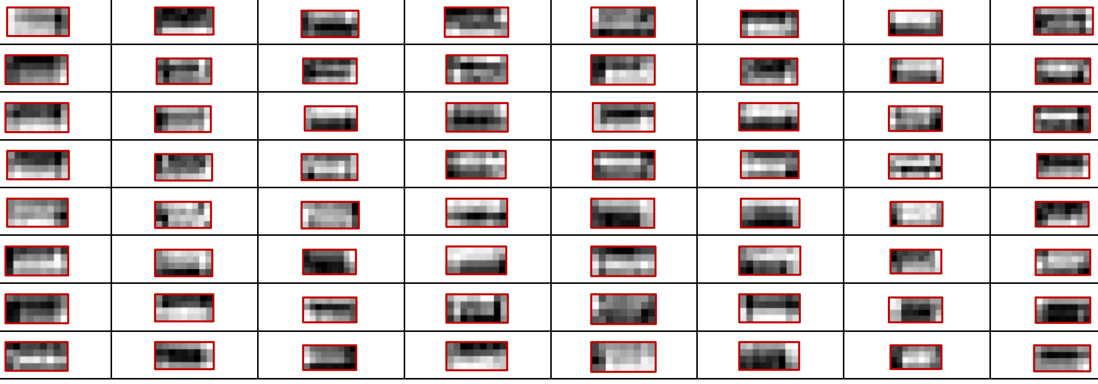
